# Sarcopenia reduces overall survival in unresectable oesophageal cancer: a systematic review and meta‐analysis

**DOI:** 10.1002/jcsm.13082

**Published:** 2022-09-24

**Authors:** Uzair M. Jogiat, Eric L.R. Bédard, Hannah Sasewich, Simon R. Turner, Dean T. Eurich, Heather Filafilo, Vickie Baracos

**Affiliations:** ^1^ Division of Thoracic Surgery, Department of Surgery University of Alberta Edmonton Alberta Canada; ^2^ School of Public Health University of Alberta Edmonton Alberta Canada; ^3^ Division of Palliative Care Medicine, Department of Oncology University of Alberta Edmonton Alberta Canada

**Keywords:** oesophageal cancer, oncology, sarcopenia, skeletal muscle index

## Abstract

Sarcopenia measured through body composition analysis is emerging as an important prognosticator among various malignancies, including oesophageal cancer. Skeletal muscle index (SMI) as determined by the third lumbar vertebrae on cross‐sectional CT images has been demonstrated as a predictor of overall survival in oesophageal cancer, using pre‐defined cut off values for sarcopenia. However, this is largely within the setting of resectable disease. The primary objective of this systematic review and meta‐analysis was to determine the effect of sarcopenia defined by SMI on overall‐survival in patients with unresectable oesophageal cancer. On 30 January 2021, a systematic search of the literature was conducted to identify the role of SMI among patients with unresectable oesophageal cancer, with overall survival as the primary outcome. Databases included MEDLINE, EMBASE, Scopus, Web of Science, and Cochrane Library. Inclusion criteria included age >18, diagnosis of oesophageal cancer, and non‐operative management. A meta‐analysis was conducted using RevMan 5.4.1 using an inverse variance, random effects model. After the removal of duplicates, 2755 unique search results were obtained. Manual screening of titles and abstracts resulted in 287 full text articles that were reviewed. Of these, five studies met the inclusion criteria with data evaluating the effect of sarcopenia defined by SMI on overall survival. A total of 783 patients, the majority of which were male (*n* = 638, 81%), with a mean age of 68 ± 2.3 years were included. 641 (82%) patients were diagnosed with squamous cell carcinoma. Sarcopenia, as determined by SMI using pre‐defined cut‐off values, was reported in 517 patients (66%). Meta‐analysis demonstrated decreased overall survival in the sarcopenia group compared with the non‐sarcopenia group (HR = 1.51; 95% CI 1.21–1.89; *P* = 0.0003; *I* ^2^ = 0%; Figure 1). No significant publication bias was noted on assessment of funnel plot and Egger's test (*P* = 0.295). Sarcopenia as defined by SMI is predictive of overall survival among patients with nonoperative oesophageal cancer. Further analysis on the effect of sarcopenia on treatment related adverse effects and complications, particularly related to chemotherapy, radiotherapy, and oesophageal stenting, is needed to identify the degree of prognostication offered by body composition analysis. Studies on the modifiability of sarcopenia will help determine the utility of nutritional interventions.

## 1 Introduction

Each year, half a million people die from oesophageal cancer, a disease characterized by an overall 5 year survival rate of 15%.^1^ Most patients present with locally advanced, unresectable, or metastatic disease at time of diagnosis.^2^ Due to dysphagia limiting caloric intake, these patients are high‐risk for malnutrition, which is closely linked to sarcopenia.^3^, ^4^ Sarcopenia is defined as the severe loss of muscle mass and function over time.^5^, ^6^, ^7^ Techniques to quantify sarcopenia have emerged through the utilization of computed tomography (CT) imaging, dual‐energy X‐ray absorptiometry (DEXA), and bioelectrical impedance analysis (BIA).^8^ With respect to CT, the skeletal muscle index (SMI) has been demonstrated as a prognostic factor in several malignancies and is a highly accessible imaging modality used for the diagnosis and staging for oesophageal cancer.^9^ The effect of sarcopenia, defined by SMI, on overall survival (OS) in patients with unresectable oesophageal cancer is unclear, with the majority of literature conducted in the setting of resectable disease.^10^, ^11^ Therefore, the primary objective of this systematic review and meta‐analysis is to investigate the effect of sarcopenia, defined by SMI, on OS, among patients with unresectable oesophageal cancer.

## 2 Methods

### 2.1 Search strategy

Using the Preferred Reporting Items for Systematic Reviews and Meta‐Analyses (PRISMA) guidelines, a systematic search of the literature was conducted in MEDLINE (1946–2021), EMBASE (1974–2021), Scopus, Web of Science, and Cochrane Library on 30 January 2021. Search terms included a combination of free‐text and controlled vocabulary terms related to oesophageal cancer and sarcopenia, including ‘Esophageal Cancer OR [variations of the term]’ AND ‘Sarcopenia OR [variations of the term]’. Refer to *Table* S1 for the full search strategy. The research team also reviewed the first 200 results in Google Scholar and the bibliography of included studies. The search results were uploaded to Covidence, an online software program allowing for the automated exclusion of duplications and manual screening of abstracts and full‐text articles.

### 2.2 Inclusion and exclusion criteria

Patients with unresectable oesophageal cancer, age ≥18, greater than five patients, sarcopenia defined by SMI, and studies published in English comprised the inclusion criteria. All other cancer types, including gastric cancer, were excluded. All study designs were included. Conference abstracts, duplicate studies, studies using the same database of patients, and studies on patients with oesophageal cancer treated with surgery were excluded. Two primary researchers evaluated titles and abstracts (U. J. and H. S.), with potentially eligible studies undergoing a full text review by two primary researchers (U. J. and H. S.). Disagreement was resolved by consensus.

### 2.3 Data extraction

Author, year of publication, study design, *n*‐values for sarcopenia and non‐sarcopenia, mean baseline patient characteristics, sarcopenia definition, adverse events to chemoradiotherapy, and OS were extracted from included studies. Baseline patient characteristics included age, sex, tumour histology, and chemotherapy or radiotherapy regimen.

### 2.4 Study outcomes

The aim of our study was to investigate the effect of sarcopenia, defined by SMI at the third or fourth lumbar vertebrae, on OS in patients with unresectable oesophageal cancer. The primary outcome measure was OS.

### 2.5 Assessment of methodological quality

Methodological quality of studies was assessed by two independent reviewers (U. J. and H. S.) using the methodological index for non‐randomized studies (MINORS) criteria. MINORS is an externally validated 12‐item index utilized to assess the methodological quality of comparative and non‐comparative studies.^12^ *Table* 1 provides the MINOR score and baseline patient characteristics. *Table* 2 provides the details of body composition methodology in the included studies.

**Table 1:** Characteristics of the included studies

Author, year	*n*	Design	Sarcopenia, *n* (%)	Age	Sex, % male	SCC %	Ad. %	MINO‐RS score
Jarvinen, 2018	238	R CS	199 (83.6)	67.52 ± 10.9^b^	66.8	46.2	44.5	18
Sato, 2018	48	R CS	34 (70.8)	S: 65.5 (41–79) N: 70.0 (53–77)	66.7	100	0	20
Gabiatti, 2019	123	R CS	57 (46.3)	59.3 ± 11.7^b^	87.7	91.1	8.9	18
Ma, 2019	198	R CS	101 (51.0)	67 (36–91)^a^	96.0	98.5	1.5	18
Onishi, 2019	176	R CS	101 (57.4)	S: 65.1 ± 6.1^b^ N: 65.3 ± 6.2^b^	85.2	100	0	18

Abbreviations: CS, cohort study; R, retrospective; S, sarcopenia; N, non‐sarcopenia; Pre‐tx, pre‐neoadjuvant therapy; Pre‐op, pre‐operative; SCC, squamous cell carcinoma; Ad., adenocarcinoma.

^a^ Range.

^b^ Standard deviation.

**Table 2:** Details on the methodology of body composition analysis of the included studies

Author, year	Region	CT level of assessment	CT muscle (HU)	Definition(s)
Jarvinen, 2018	Europe	L3 MP	−29 to +150	Sarcopenia M ≤ 55 cm^2^/m^2^; F ≤ 39 cm^2^/m^2^
Sato, 2018	Asia	L3	−29 to +150	Sarcopenia: M < 52.4 cm^2^/m^2^; F < 38.5 cm^2^/m^2^
Gabiatti, 2019	South America	L3	−29 to +150	Sarcopenia: M, L < 43 m^3^/m^2^, H < 53 cm^2^/m^2^; F < 41 cm^2^/m^2^
Ma, 2019	Asia	L3	−29 to +150	Sarcopenia: M < 49 cm^2^/m^2^; F < 31 cm^2^/m^2^
Onishi, 2019	Asia	L3	−29 to +150	Sarcopenia: M < 52.4 cm^2^/m^2^; F < 38.5 cm^2^/m^2^

*Note*: All studies normalized measurements.

Abbreviations: F, female; M, male; HU, Hounsfield Units; H, BMI > 25 kg/m^2^; L, BMI < 25 kg/m^2^; L3, third lumbar vertebrae; MP, midpoint.

### 2.6 Statistical analysis

Meta‐analysis was conducted to evaluate the difference in OS in patients with sarcopenia compared with patients without sarcopenia. Estimated effects were calculated using RevMan 5.4.1 software with an inverse variance random‐effects model for OS. Heterogeneity was quantified by the *I* ^2^ statistic: (1) low <25%; (2) moderate = 25–75%; (3) high >75%. Tests for statistical significance were two‐tailed with significant *P*‐values defined as <0.05. Publication bias was assessed by funnel plot generated in Stata SE 17.0. The Egger test was performed to assess funnel plot asymmetry. Leave‐one‐out sensitivity analysis was conducted by sequentially removing each study to determine the effect of heterogeneity.

## 3 Results

### 3.1 Study selection

**Figure 1:**
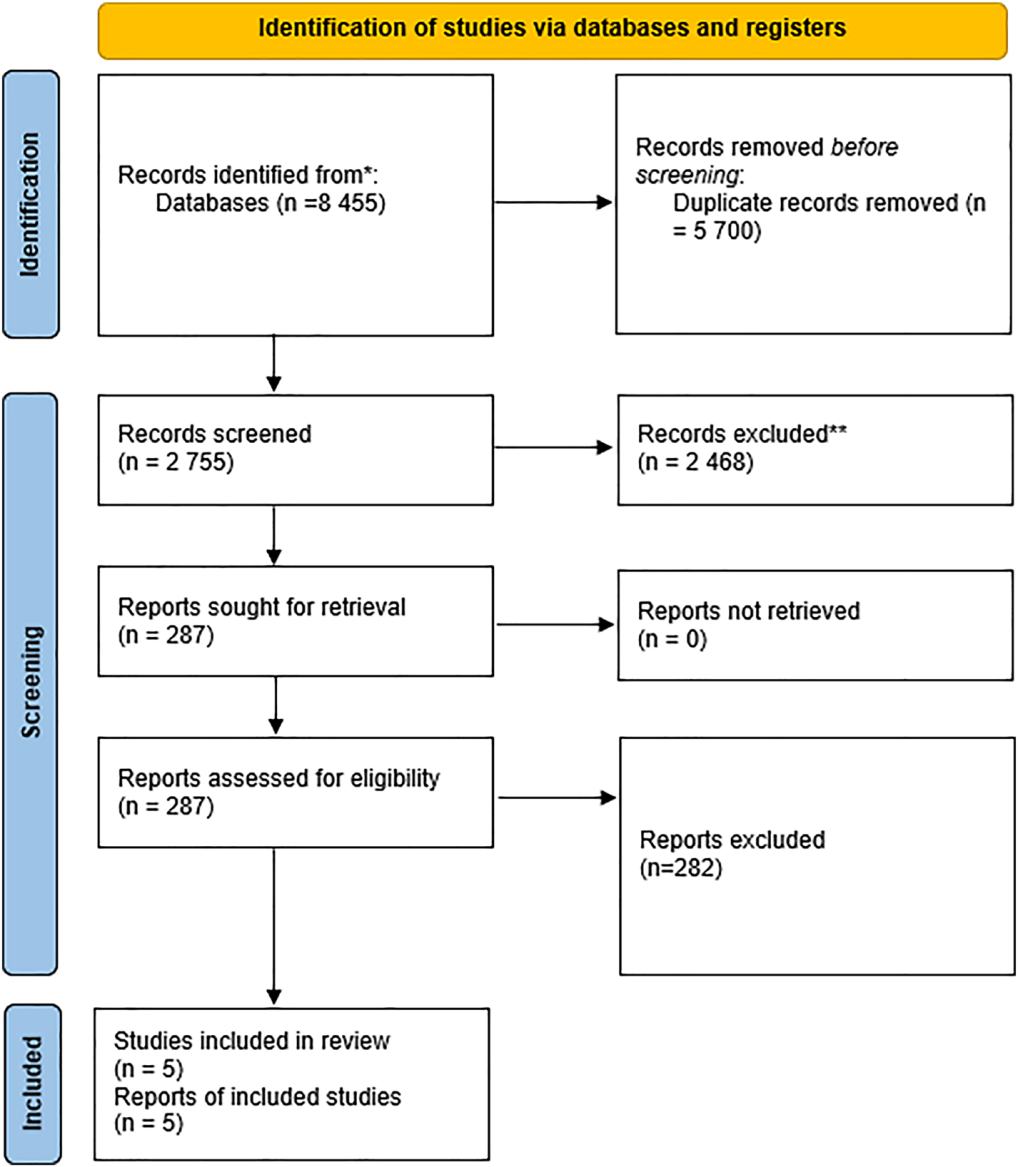
Preferred reporting items for systematic review and meta‐analyses (PRISMA) diagram of included studies.

A systematic search rendered 8455 studies, with exclusion of duplicates, resulting in 2755 unique records. Of these, 2468 abstracts were excluded for not meeting the inclusion criteria or meeting an exclusion criterion. This resulted in 287 full‐text articles that were reviewed. Of these, five studies met the inclusion criteria, with data evaluating the effects of sarcopenia defined by SMI on overall survival. All five articles were retrospective. *Figure* 1 is the PRISMA flow diagram demonstrating the results of the systematic search.

### 3.2 Study characteristics

A total of 783 patients, the majority of which were male (*n* = 638, 81%), with a mean age of 68 ± 2.3 years were included (*Table* 1). 641 (82%) patients were diagnosed with squamous cell carcinoma. Sarcopenia was reported in 517 patients (66%). Two studies reported data on adverse events of chemotherapy stratified by sarcopenia status, with no significant difference observed between the groups.^13^, ^14^ Overall survival was reported in all five studies.^13^, ^14^, ^15^, ^16^, ^17^

### 3.3 Critical appraisal of studies

Mean MINORS scores of the included studies was 18.4 ± 0.8, indicating methodological adequacy (*Table* 1). Interstudy variability was encountered in the chemotherapy and radiation regimens, as well as the definitions for sarcopenia (*Table* 2). Cut‐off variables were reported in all of the included studies. Median follow‐up ranged from 9 to 38 months.

### 3.4 Reduced overall survival in patients with sarcopenia

**Figure 2:**
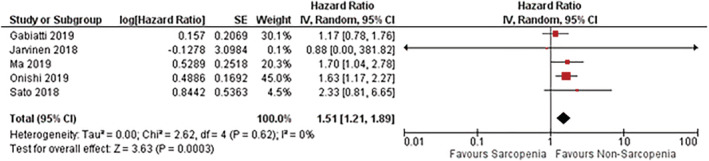
Summary meta‐analysis of studies reporting the effect of sarcopenia, defined by SMI, on overall survival in patients with unresectable oesophageal cancer.

The effect of sarcopenia defined by SMI on OS was reported in five studies included in this meta‐analysis. In patients with unresectable oesophageal cancer, meta‐analysis revealed a significant reduction in OS among patients with sarcopenia compared with those without sarcopenia (HR 1.51; 95% CI: 1.21–1.89; *P* = 0.0003; Figure 2). Studies exhibited low heterogeneity (*I* ^2^ = 0%), and on sensitivity analysis, Jarvinen *et al*. was identified as the major contributor to encountered heterogeneity; however, removal of this study from the analysis had no effect on the statistical significance of the pooled results.^17^ No significant publication bias was noted on assessment of funnel plot and Egger's test (*P* = 0.295).

A sub‐group analysis was conducted on studies including patients who exclusively underwent definitive chemoradiotherapy. The effect of sarcopenia defined by SMI on OS was reported in three studies in this sub‐group. In patients with unresectable oesophageal cancer who underwent definitive chemoradiotherapy, meta‐analysis revealed a significant reduction in OS among patients with sarcopenia compared with those without sarcopenia (HR 1.44; 95% CI: 1.04–1.99; *Figure* S1). Studies exhibited low heterogeneity (*I* ^2^ = 10%). No major contributors to heterogeneity were identified on sensitivity analysis, and no significant publication bias was noted on assessment of funnel plot and Egger's test (*P* = 0.213).

## 4 Discussion

Sarcopenia defined by SMI is a significant predictor of OS in patients with unresectable oesophageal cancer. The strengths of this systematic review and meta‐analysis include the adherence to PRISMA guidelines and the in‐depth critical appraisal of the included studies. Although there have been a number of systematic reviews and meta‐analyses conducted on sarcopenia in oesophageal cancer, to our knowledge, these have been conducted in the setting of resectable disease.^8^, ^18^, ^19^, ^20^

Patients with unresectable oesophageal cancer generally have a poor prognosis.^1^ This is secondary to the aggressive nature of the malignancy, as well as the lack of effective medical therapies.^21^ Understanding how sarcopenia impacts prognosis can provide valuable information to patients and their medical providers. Although the current literature has not determined the reversibility of sarcopenia, as of yet, the identification of sarcopenic patients can assist in risk stratification models. Owing to a multitude of tumour, patient, and treatment related‐factors, survival in the setting of unresectable oesophageal cancer is heterogenous.^22^ Sarcopenia may serve as an additional factor to consider when assessing survival in this patient population.

The decision to define sarcopenia by SMI exclusively was based on several factors. First of all, selecting a single body composition parameter limits study heterogeneity. Selecting a CT‐based parameter is logical given the widespread availability and utilization of this imaging modality for the staging and diagnosis of oesophageal cancer. Other modalities such as BIA and DEXA are not widely used‐clinically, and the selection of BMI alone is not appropriate given its poor reflection of body composition.^23^ Although psoas‐muscle index has been used as a body composition parameter in some studies, we did not select this parameter due the lack of data validating a single‐muscle approach to be reflective of whole‐body mass and given that sarcopenia is a systemic process.^24^, ^25^

The results of our sub‐group analysis on patients who underwent definitive chemoradiotherapy demonstrate a similar effect size when compared with the entire meta‐analysis comprising two additional studies including patients with metastatic oesophageal cancer. Given that sarcopenia is classified at the staging CT scan in the majority of studies included in the meta‐analysis, the results suggest that the prognostic impact of sarcopenia is present throughout disease progression. This notion is corroborated by a recently published systematic review on sarcopenia in resectable oesophageal cancer.^26^

Limitations to our study include the heterogeneity among the included studies regarding the type of medical therapy received, endoscopic stenting, and definition of unresectable disease. Furthermore, squamous cell carcinoma was the predominant tumour histology of the included studies, accounting for the vast majority of patients. This is likely secondary to the majority of studies being conducted in an Asian population.^27^ As adenocarcinoma is more commonly associated with obesity, there may exist unique implications on sarcopenia based on tumour histology that are not captured by this review.^28^ It is important to recognize that squamous cell carcinoma likely represents a completely different disease process in comparison with adenocarcinoma, complicated by worse prognoses and more aggressive disease.^29^ The included studies did not stratify statistical analyses by tumour histology; thus, these differences are not captured by the meta‐analyses.

Given the high heterogeneity attributed to Jarvinen *et al*. in the sensitivity analysis, it is worth examining further where these differences arise.^17^ The sarcopenia cut‐off values for SMI utilized in this retrospective cohort study differ from the other studies included in the meta‐analysis and are based on the reference values from Fearon *et al*., an international Deplhi consensus statement on the classification of cancer cachexia.^30^ These reference values were derived from a population of obese patients, however, and may not be representative of the population examined by Jarvinen *et al*., with a mean BMI of 21.7 kg/m^2^ (19.2–24.5). This is particularly relevant, as the key findings of the study are paradoxical in that sarcopenia was not significantly associated with overall survival in the Kaplan–Meier survival analysis (log‐rank *P* = 0.61), whereas the SMI, independent of sarcopenia status, was significantly associated with overall survival in the cox proportional hazards model (HR 0.98, 95% CI 0.97–0.99, *P* = 0.033). Although these findings may be attributed to potential misclassification of sarcopenia status, it is important to consider that all patients included in the analysis received oesophageal stenting. The potential for this intervention to serve as an effect modifier in the relationship between sarcopenia and survival has yet to be determined, and is therefore, an important consideration when interpreting the study's findings. Lastly, the evaluation of sarcopenia was based on the CT scan obtained 2 weeks post‐stent insertion, implying advanced disease, whereas the staging CT scan was most often used in the other studies included in the meta‐analysis. Sarcopenia changes over time with respect to disease progression, and thus, this likely also contributes to the difference in findings observed between Jarvinen *et al*. and the other studies included in the meta‐analysis.

Given the lack of data on drug‐limited toxicities and adverse events of chemoradiotherapy, we were unable to conduct data analysis on this outcome. This is particularly relevant given that most chemotherapy drugs are dosed based on weight, independent of sarcopenia status.^31^ Many of these medications undergo metabolization in lean tissue compartments, which are influenced by sarcopenia status.^32^ Therefore, this could lead to potential supra‐therapeutic dosing, leading to increased rates of drug‐limited toxicities, and is an area of future research.

## 5 Conclusion

This systematic review and meta‐analysis highlights the prognostic value of sarcopenia on OS in patients with unresectable oesophageal cancer. This finding supports the incorporation of sarcopenia status in the evaluation of prognosis in this patient population. Studies on the assessment of pre‐habilitation programmes focused on targeted nutritional interventions and identification of nutritional guidelines by body composition are required to determine the modifiability of this prognostic factor.

## Conflicts of interest

The authors declare no conflicts of interest. The authors have no financial relationships relevant to this study. Dr. Turner declares a financial relationship with Astra Zeneca and Ethicon.

## Supporting information


**Figure S1:** Summary meta‐analysis of sub‐group analysis reporting the effect of sarcopenia, defined by SMI, on overall survival in patients with unresectable oesophageal cancer who received definitive chemoradiotherapy.
