# Supplementary material for: Sarcopenia reduces overall survival in unresectable oesophageal cancer: a systematic review and meta‐analysis
Source: J Cachexia Sarcopenia Muscle. 2022 Sep 24;13(6):2630–6. doi: 10.1002/jcsm.13082 (PMC9745498; doi:10.1002/jcsm.13082)
Supplement: Supplementary file 1 — Figure S1: Summary meta‐analysis of sub‐group analysis reporting the effect of sarcopenia, defined by SMI, on overall survival in patients with unresectable oesophageal cancer who received definitive chemoradiotherapy. [file JCSM-13-2630-s001.docx]

**
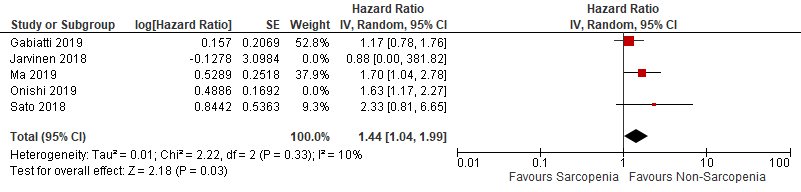
**

Supplementary Figure 1: Summary meta-analysis of sub-group analysis reporting the effect of sarcopenia, defined by SMI, on overall survival in patients with unresectable esophageal cancer who received definitive chemoradiotherapy.
